# Epidemiologic features of enterovirus associated with hand, foot and mouth disease in 2013 and 2014 in Shenzhen, China

**DOI:** 10.1038/s41598-019-40402-2

**Published:** 2019-03-07

**Authors:** Kelin Xiao, Lian Duan, Yun Peng, Maocai Wu, Guangxing Mai, Zehao Yan, Shuiwen Chen, Yihan Lu

**Affiliations:** 1 International institute of Infection and Immunity, Shantou University Medical College, Shantou, 515041 China; 2 Central Laboratory, Maternal-Fetal Medicine Institute, Baoan Maternal and Child Health Hospital, Jinan University, Shenzhen, 518102 China; 3 Shenzhen liver diseases institute, Shenzhen Third People’s Hospital, Shenzhen, 518112 China; 4 Department of pediatrics, Baoan Maternal and Child Health Hospital, Jinan University, Shenzhen, 518102 China; 5 Department of Epidemiology, The Key Laboratory of Public Health Safety of Minister of Education, School of Public Health, Fudan University, Shanghai, 200032 China

## Abstract

Hand, foot and mouth disease (HFMD) is responsible for a heavy economic and social burden in the Asia-Pacific region. Previous studies have shown that coxsackievirus A6 (CVA6) and coxsackievirus A10 (CVA10) have become the predominant agents of HFMD in mainland China in recent years, replacing enterovirus 71 (EV71) and coxsackievirus A16 (CVA16), although it is unclear if this is consistent throughout China. In this study, samples from 253 HFMD cases were collected in Shenzhen, China, from May 2013 through April 2014 to identify the etiological agent of HFMD. In total, 64.8% (164/253) of HFMD cases were enterovirus positive, in which 81.1% (133/164) were determined to be CVA6. The phylogenetic tree of the partial viral protein 1 sequence showed that the CVA6 isolates were divided into four clusters (Clusters A to D), and cluster D was further divided into four sub-clusters (Clusters D1 to D4). The 133 CVA6 samples isolated in our study were classified into cluster D4, in which the first identified sequence was isolated in Shenzhen in 2008. This study demonstrated that the CVA6 cluster D4, which is predominantly circulating in HFMD in mainland China, may have originated from a local strain identified in 2008 in Shenzhen.

## Introduction

Hand, foot and mouth disease (HFMD) is a common communicable disease in children under 5 years old. Typical manifestations of HFMD include fever, vesicular rash in hand, foot, and mouth^1^. HFMD outbreaks have been reported in Japan, Australia, Malaysia, Singapore, Vietnam, Hong Kong, Taiwan, and mainland China since the 1970 s^2-11^. As a result, it has become a public health concern with a heavy economic burden across the world^12^. In 2008, a large outbreak of HFMD occurred in China that involved 6,049 clinically diagnosed cases including 22 deaths^9^.

HFMD cases are mostly mild and self-limited. However, it is difficult to distinguish severe and mild cases at an early stage, since very few cases have obvious symptoms. Previous studies reported that severe HFMD cases are mostly caused by enterovirus 71 (EV71), which has been proven to be associated with severe manifestations, such as pulmonary edema, brain stem encephalitis, acute flaccid paralysis (AFP), meningoencephalitis, aseptic meningitis, and cerebellitis^11,13–16^. Therefore, it is crucial to determine the serotype of enterovirus to identify potentially severe HFMD cases.

EV71 and coxsackievirus A16 (CVA16) had been confirmed to be the most common agents of HFMD in China when HFMD surveillance initiated in 2008^16–19^. However, the most prevalent serotypes of enterovirus have shifted to coxsackievirus A6 (CVA6) and A10 (CVA10) in China starting in 2012^20,21^. Additionally, CVA6 and CVA10 have been responsible for HFMD outbreaks in Finland (2008) and France (2010)^22,23^. These studies indicate that the epidemic trend of enterovirus may be shifting. Therefore, from May 2013 through April 2014, we conducted a study in a tertiary maternal and child hospital in Shenzhen, China to determine the serotype distribution of enterovirus in HFMD cases and to further study the phylogenetics of CVA6.

## Results

### HFMD cases

A total of 253 HFMD cases were included in our study, in which 83.8% (212/253) were diagnosed between June and September 2013. There were 161 male and 92 female cases, with a male-to-female ratio of 1.75. The median age of cases was 1 year old, ranging from 4 months to 23 years old. The majority (98.4%, 249/253) were under 6 years old, except one 14-year-old teenager and one 23-year-old nursing mother.

### Clinical presentation

The typical clinical presentation included fever, rash and vesicles. In total, 81.0% (205/253) of the cases had a fever and 22.5% (57/253) had a high fever (≥39 °C). Rash was intraoral (48.6%, 123/253), on the limbs (99.6%, 252/253), on the buttocks (67.2%, 170/253), on the trunk (24.1%, 61/253), and on the head and face (7.9%, 20/253). The majority of the cases (96.8%, 245/253) had a rash accompanied by vesicles. Additionally, a total of 13 cases reported skin itchiness (Table 1).

**Table 1:** Demographics and manifestations of the 253 HFMD cases included in the study.

	CVA6	EV71	CA16	other enterovirus	non-enterovirus
Cases (%)	133 (52.6)	11 (4.3)	13 (5.1)	7 (2.8)	89 (35.2)
Male/female ratio	1.9 (87/46)	1.8 (7/4)	1.6 (8/5)	1.3 (4/3)	1.6 (55/34)
Age (year), mean ± SD	1.47 ± 1.16^a^	2.91 ± 1.30	1.54 ± 1.17	2.42 ± 1.63	1.59 ± 1.10
Fever ( ≥ 39 °C) (%)	28.6 (38/133)	0.0 (0/11)	0.0 (0/13)	14.3 (1/7)	20.2 (18/89)
Intraoral rash (%)	48.9 (65/133)	36.4 (4/11)	53.8 (7/13)	71.4 (5/7)	47.2 (42/89)
Rash on limbs (%)	99.2 (132^b^/133)	100.0 (11/11)	100.0 (13/13)	100.0 (7/7)	100.0 (89/89)
Rash on trunk (%)	27.8 (37/133)	9.1 (1/11)	0.0 (0/13)	0.0 (0/7)	25.8 (23/89)
Rash on buttocks (%)	71.4 (95/133)	72.7 (8/11)	92.3 (12/13)	57.1 (4/7)	57.3 (51/89)
Rash on head and face (%)	9.8 (13/133)	0.0 (0/11)	0.0 (0/13)	14.3 (1/7)	6.7 (6/89)
Vesicles (%)	97.8 (130/133)	100.0 (11/11)	100.0 (13/13)	100.0 (7/7)	94.4 (84/89)
Skin itchiness (%)	7.5 (10/133)	0.0 (0/11)	0.0 (0/13)	0.0 (0/7)	3.4 (3/89)

^a^Two older HFMD cases (14-year-old and 23-year-old) were excluded.

^b^One case had an intraoral rash and rash on trunk and buttocks.

### Enterovirus serotyping

Overall, 64.8% (164/253) of the HFMD cases were enterovirus positive. Among male cases, it was 65.8% (106/161), compared to 63.0% (58/92) in female cases (*P* = 0.61) (Table 2). All the cases were divided into five groups by age. There were only 4 cases in those ≥6 years old. In the other four age groups, the prevalence of enterovirus varied between 53.8% and 67.5% (*P* = 0.46) (Table 2).

**Table 2:** Enterovirus serotypes by sex and age.

	No. cases	No. enterovirus-positive cases (%)	No. enterovirus serotypes (%)						
			CVA6^a^	EV71^b^	CVA16	CVA9	CVA10	CVB2	Undetermined^c^
**Sex**									
male	161	106 (65.8)	87 (82.1)	7 (6.6)	8 (7.6)	1 (0.9)	2 (1.9)	1 (0.9)	0
female	92	58 (63.0)	46 (79.3)	4 (6.9)	5 (8.6)	0	0	0	3 (5.2)
**Age (year)**									
<1	53	34 (64.2)	30 (88.2)	0	3 (8.8)	0	1 (3.0)	0	0
1	114	77 (67.5)	67 (87.0)	2 (2.6)	6 (7.8)	0	1 (1.3)	1 (1.3)	0
2	39	21 (53.8)	16 (76.2)	2 (9.5)	2 (9.5)	1 (4.8)	0	0	0
3–5	43	29 (67.4)	17 (58.6)	7 (24.1)	2 (6.9)	0	0	0	3 (10.4)
≥6	4	3 (75.0)	3 (100.0)	0	0	0	0	0	0

^a^Cases under 3 years old vs. cases 3–5 years old, *P* = 0.001.

^b^Cases under 3 years old vs. cases 3–5 years old, *P* = 0.001.

^c^Undetermined serotype by VP1 nested PCR.

Of the 164 enterovirus positive cases, 161 (98.2%) were determined based on an examination of a 336-nt partial viral protein 1 (VP1) sequence, whereas the other 3 cases were undetermined (Table 3). A total of six enterovirus serotypes were determined in our study. CVA6 was the predominant serotype, followed by CVA16 and EV71 (Table 3).

**Table 3:** Enterovirus serotypes by calendar time.

	No. enterovirus-positive cases	No. enterovirus serotypes (%)						
		CVA6	EV71	CVA16	CVA9	CVA10	CVB2	Undetermined
May, 2013	9	6 (66.7)	1 (11.1)	0	1 (11.1)	0	1 (11.1)	0
Jun, 2013	14	8 (57.1)	1 (7.1)	2 (14.3)	0	1 (7.1)	0	2 (14.3)
Jul, 2013	39	36 (92.3)	1 (25.6)	2 (51.3)	0	0	0	0
Aug, 2013	43	42 (97.7)	0	0	0	1 (2.3)	0	0
Sep, 2013	37	36 (97.3)	1 (2.7)	0	0	0	0	0
Oct, 2013	6	4 (66.7)	0	2 (33.3)	0	0	0	0
Nov, 2013	1	1 (100.0)	0	0	0	0	0	0
Dec, 2013	1	0	0	1 (100.0)	0	0	0	0
Mar, 2014	8	0	5 (62.5)	3 (37.5)	0	0	0	0
Apr, 2014	6	0	2 (33.3)	3 (50.0)	0	0	0	1 (16.7)
Total	164	133 (81.1)	11 (67.1)	13 (7.9)	1 (0.6)	2 (12.2)	1 (0.6)	3 (1.8)

The prevalence of CVA6 in the male cases (82.1%, 87/106) and female cases (79.3%, 46/58) was similar (*P* = 0.67) (Table 2). Furthermore, CVA6 was also predominant across all five age groups. However, it was significantly higher among cases under 3 years old (85.6%, 113/132) than among cases 3–5 years old (58.6%, 17/29) (*P* = 0.001). Additionally, a significantly higher prevalence of EV71 was observed among cases 3–5 years old (24.1%, 7/22), compared to cases under 3 years old (3.0%, 4/132) (*P* = 0.001).

### Phylogenetics of CVA6

**Figure 1:**
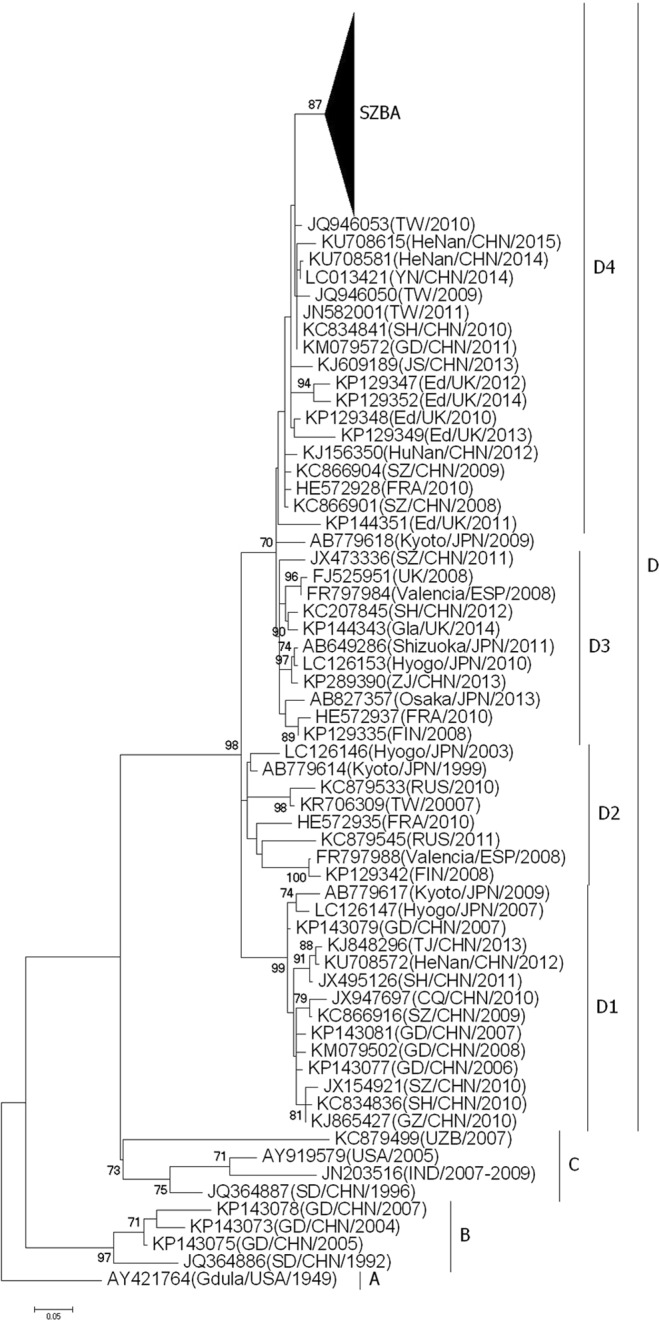
Phylogenetic tree reconstructed based on a 336-nt CVA6 partial VP1 sequence. The tree was reconstructed using the maximum likelihood method (bootstrap of 1000 replications) in MEGA 6.0. SZBA: a total of 133 CVA6 partial sequences isolated in our study.

A total of 133 CVA6 366-nt partial VP1 sequences isolated in our study and 1212 sequences retrieved from GenBank were combined together for further phylogenetic analysis. The phylogenetic tree showed that CVA6 was divided into four clusters (A to D). Cluster A contained only one strain isolated in the USA in 1949, which could be the origin of HFMD. Several strains isolated between 1992 and 2007 in mainland China formed cluster B. Similarly, cluster C contained only strains isolated in mainland China, India, and the USA during 1996–2009. Cluster D was the largest one that was further divided into four sub-clusters (D1 to D4), suggesting gradual expansion and dissemination of HFMD in recent years, especially in mainland China. All of the 133 CVA6 sequences obtained in our study were classified into cluster D4 (Fig. 1 and Fig. S1).

## Discussion

HFMD has a typical seasonality that is associated with meteorological factors, such as temperature, humidity, sunshine, and air pressure^16,24,25^. The peak of HFMD incidence usually occurs in summer. However, prevalent seasons of HFMD in Shenzhen are both summer and fall. Additionally, it is basically active throughout the year in Shenzhen, which is consistent with seasonal patterns reported in Hong Kong^26^, Beijing^27^ and Thailand^28^.

The clinical presentation of HFMD cases caused by CVA6 was fever and rash on limbs, buttocks and intraoral areas, and 97.8% cases in this study had vesicles. Additionally, CVA6 cases were more likely to have fever (≥39 °C) and rash on trunk, head and face, compared to cases with EV71 and CVA16, as has been previously reported^20,22,28,29^.

In our study, 64.8% of the HFMD cases were enterovirus positive, which were determined by using a semi-nested PCR serotyping method^30^. The findings showed that CVA6 was the predominant serotype, accounting for approximately 81% of enterovirus positive cases, which was much higher than the prevalence of EV71 (7.9%) and CVA16 (6.7%). CVA6 was first reported to cause HFMD outbreaks in Finland in fall 2008^31^, with following reports in Singapore in 2008^8^, France^22^ and Taiwan^32^ in 2010, USA^33^, Spain^34^, Japan^35^, and Cuba^36^ in 2011, and Thailand^28^ in 2012. In mainland China, CVA6 was a minor contributor to HFMD for a long time; however, a dramatic increase of this serotype was found in HFMD cases since fall 2012^20,37^. Our study has also illustrated that enterovirus serotypes in Shenzhen had shifted by 2013; CVA6 had taken the place of both EV71 and CVA16, and had become the predominant serotype in HFMD cases, which confirmed previous studies^21,37,38^. He *et al*. reported the variation of enterovirus serotypes from 2008 through 2012 in Shenzhen and suggested that CVA6 and CVA10 had gradually become the most common enterovirus serotype in Shenzhen starting in 2012^21^, and Lu *et al*. reported that CVA6 was a new epidemic trend in Guangdong province, where Shenzhen is located, as of late 2012^37^.

The majority (98.4%) of cases in our study were less than 6 years old. The prevalence of enterovirus was not significantly different across age groups. Additionally, we compared the distribution of enterovirus serotype across age groups and noticed that CVA6 was the most common serotype regardless of age groups. However, the proportion of CVA6 among cases under 3 years old was significantly higher than that among cases 3–5 years old; instead, EV71 was significantly higher. It indicated that EV71 preferentially infects preschool-aged children, although CVA6 remains most common^20^. We also compared the difference of enterovirus infection and distribution of serotypes by gender; the findings showed no statistically significant difference between male and female cases. Furthermore, CVA6 was the most common serotype in both male and female cases, suggesting that gender is not related to enterovirus infection or serotype^20^.

In this study, we explored the origin of CVA6 with phylogenetic analysis. All 133 sequences isolated in our study belonged to cluster D4, which has been reported to be circulating in United Kingdom and France. The cluster contained isolates in multiple provinces in mainland China, such as Shanghai, Yunnan, Henan, Jiangsu, Shandong, and Guangdong. Our findings demonstrated that CVA6 within cluster D4 has become the prevalent serotype in mainland China and has spread into Europe. Furthermore, the phylogenic tree has shown that cluster D4 was first isolated in Shenzhen in 2008 (GenBank Accession No. KC866901) and continually circulated in mainland China through 2015 (Fig. 1 and Fig. S1). The cluster was close to strains isolated in Finland in 2008; however, it remained separate from strains isolated in other countries, suggesting that CVA6 within cluster D4 might originate from a local strain, which has been documented in previous studies^39,40^.

In conclusion, our study demonstrated that CVA6 has become predominant in HFMD cases. Cluster D4 of CVA6 originated in Shenzhen and has been circulating in mainland China since 2008.

## Methods

### Participants

From May 2013 through April 2014, a total of 253 HFMD cases were included from the Department of Paediatrics and the Department of Dermatology in Baoan Maternal and Child Health Hospital, Jinan University, Shenzhen, China. Manifestations of the cases included eruption rash and/or vesicles on the hand, foot, buttock or trunk. Diagnosis criteria of HFMD are defined according to the National Health and Family Planning Commission of the People’s Republic of China (http://www.nhfpc.gov.cn/jkj/s3577/200805/e73df45b7b1549188b1d4e1efd604da9.shtml). This study was approved by the Ethics Committee of Baoan Maternal and Child Health Hospital, Jinan University, Shenzhen, China. All the examinations were carried out per official guidelines and regulations. Informed consent was obtained from the patients’ parents or legal guardians.

### Enterovirus examination

Vesicular fluid, throat swab, stool or anal swabs of cases were collected for enterovirus examination. A total of 253 specimens were collected, one from each HFMD case, by sterile swabs and then put into sterile containers. Specimens were immediately sent to a laboratory and stored in −20 °C prior to examination. All specimens were examined for enterovirus by using a pan-enterovirus 5′ UTR one-step reverse transcript real-time quantitative PCR (RT-qPCR) Kit (Da’an gene Comp. LTD, Guangzhou) according to the manufacturer’s instructions. Briefly, specimens were dissolved in 1 mL of normal saline and then centrifuged at 3500 *g* for 5 min. A total of 350 μL of supernatant was drawn for RNA extraction using a commercial kit containing TRIzol (Da’an gene Co. Ltd, Guangzhou). One-step RT-qPCR was then performed in the MJ Research Opticon 2^TM^ system (BIORAD, USA).

### Enterovirus serotyping

The specimens that were enterovirus positive were further serotyped using a viral protein 1 (VP1) semi-reverse transcription nested PCR, which can identify almost all enterovirus serotypes^30^. The cDNA was synthesized using PrimeScript^TM^ II 1st strand cDNA synthesis kit (Takara Biotechnology (Dalian) Co.,Ltd). A 25 µL volume of PCR mix was prepared as follows: 2.5 µL of 10 × PCR buffer (Qiagen), 1.0 µL of MgCl_2_ (25 mM), 1.0 µL of dNTP (2.5 mM), 1.0 µL of each forward and reverse primers (10 mM; Primers 222 and 224 for the first round, primers AN88 and AN89 for the second round), 0.1 µL of HotStar *Taq* polymerase (5 U/µL, Qiagen), 3 μL of template DNA (cDNA for the first round and products of the first round PCR for the second round), and molecular biology-grade water. The thermal profile in both sets of PCR was 95 °C for 15 min, 35 cycles of 95 °C for 1 min, 50 °C for 1 min, and 72 °C for 1 min, then 72 °C for 10 min. PCR products were sequenced in both directions by using the BigDye Terminator (version 3.1) cycle sequencing kit and the ABI PRISM 3730xl DNA analyzer (Thermofisher Sientific, USA). Enterovirus serotype was determined with the BLAST search in GenBank.

### Phylogenetic analysis

CVA6 sequences were aligned by using BioEdit 7.0 software. A phylogenetic tree was reconstructed by maximum likelihood method with a GTR + G + I model (bootstrap test of 1000 replications) in MEGA 6.0. A total of 1345 CVA6 sequences, including 133 sequences isolated in our laboratory and 1212 sequences retrieved from GenBank, were combined to reconstruct a phylogenetic tree. Subsequently, a smaller phylogenetic tree was reconstructed with the 133 sequences in our laboratory and 61 representative sequences (out of the 1212) from GenBank. The 133 CVA6 partial VP1 sequences were deposited into GenBank under the following accession numbers: MG195737 to MG195869.

### Statistical analysis

Chi-square tests or Fisher’s exact tests were employed to compare differences between groups using SPSS17.0 software (IBM, USA). A *P* value of less than 0.05 was considered to be statistically significant.

## Supplementary information

Figure S1. Phylogenetic analysis of CVA6 from HFDM cases in Shenzhen based on partial VP1 sequences (nt 2573 to 2908 according to Gdula/USA/1949 strain AY421764).
