# Supplementary material for: Epidemiologic features of enterovirus associated with hand, foot and mouth disease in 2013 and 2014 in Shenzhen, China
Source: Sci Rep. 2019 Mar 7;9:3856. doi: 10.1038/s41598-019-40402-2 (PMC6405776; doi:10.1038/s41598-019-40402-2)
Supplement: Supplementary file 1 — Figure S1. Phylogenetic analysis of CVA6 from HFDM cases in Shenzhen based on partial VP1 sequences (nt 2573 to 2908 according to Gdula/USA/1949 strain AY421764). [file 41598_2019_40402_MOESM1_ESM.pdf]

Epidemiologic features of enterovirus associated with hand, foot and mouth disease in 2013 and 2014 in Shenzhen, China

Kelin Xiao[1,2†], Lian Duan[3†], Yun Peng[3], Maocai Wu[3], Guangxing Mai[2], Zehao Yan[2], Shuiwen Chen[4]*, Yihan Lu[5]*.

† These authors contributed equally to the work.

[1] International institute of Infection and Immunity, Shantou University Medical College, Shantou 515041, China

[2] Central Laboratory, Maternal-Fetal Medicine Institute, Baoan Maternal and Child Health Hospital, Jinan University, Shenzhen 518102, China

[3] Shenzhen liver diseases institute, Shenzhen Third People's Hospital, Shenzhen 518112, China

[4] Department of pediatrics, Baoan Maternal and Child Health Hospital, Jinan University, Shenzhen 518102, China

[5] Department of Epidemiology, The Key Laboratory of Public Health Safety of Minister of Education, School of Public Health, Fudan University, Shanghai 200032, China

Running title: Coxsackievirus A6 circulating in Shenzhen, China

Keywords: hand-foot and mouth disease (HFMD); enterovirus; serotype; coxsackievirus A6 (CVA6); phylogenetics.

Category: Viorology or Epidemiology

***Author for correspondence:** Dr. Shuiwen Chen

Email: csw319@163.com

Address: Level 4, Department of pediatrics, Baoan Maternal and Child Health Hospital, Jinan University, No.56, Yulv road, Baoan, Shenzhen 518102, China.

Phone: +86 755 27863999 Fax: +86 75527834718

***Author for correspondence:** Dr. Yihan Lu

Email: luyihan@fudan.edu.cn

Address: Fosun Tower Room 513, 131 Dong'an Road , Fudan University School of Public Health, Shanghai 200032, China

Phone: +86 2154237088 Fax: +86 21 54237088

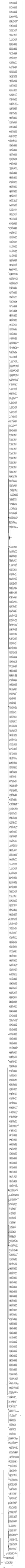

Supplemental figure legend

Figure S1. Phylogenetic analysis of CVA6 from HFDM cases in Shenzhen based on partial VP1 sequences (nt 2573 to 2908 according to Gdula/USA/1949 strain AY421764).

In total, 133 CVA6 sequences isolated from our lab (SZBA) and 1212 others sequences downloaded from Genbank, were used to construct phylogentic tree. Phylogenetic tree was constructed by using Mega 6.0, the maximum-likelihood (ML) methods were used for analyzed the phylogenies of CVA6 and bootstrap with 1000 replicates.
